# Supplementary material for: Inhibition of complement C3 prevents osteoarthritis progression in guinea pigs by blocking STAT1 activation
Source: Commun Biol. 2024 Mar 27;7:370. doi: 10.1038/s42003-024-06051-6 (PMC10973449; doi:10.1038/s42003-024-06051-6)
Supplement: Supplementary file 2 — Supplementary Information [file 42003_2024_6051_MOESM2_ESM.pdf]

Supplementary Fig 1

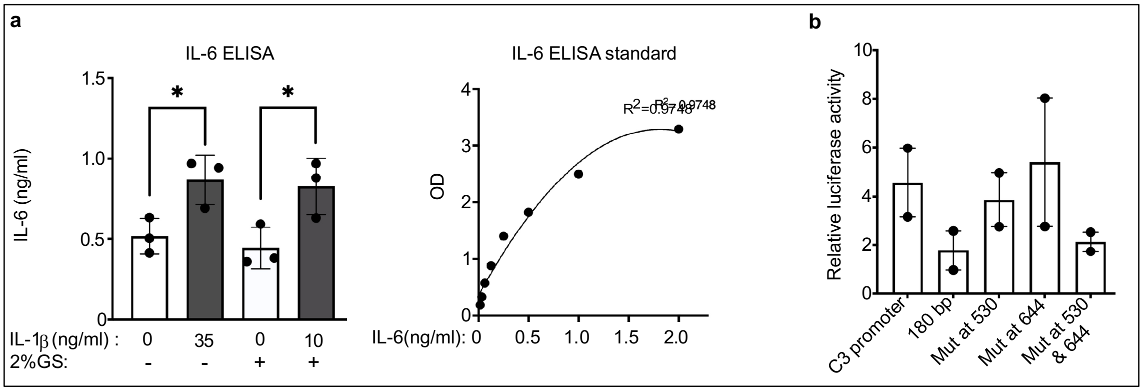

Fig. S1 a The amount of IL-6 in culture conditioned media was measured by ELISA after 10 or 35 ng/mL of IL-1? stimulation in primary human articular chondrocytes. The error bars represent standard error of mean. The significance was determined by unpaired two-tailed t-test (n= 3 p= 0.0325, p= 0.0382). b the Cypridina luciferase/red firefly luciferase dual report system was used to analyze the activity of C3 promoter with different mutations in its potential STAT1 binding sites in human primary articular chondrocytes. The error bars represent standard error of mean.

Original images

Fig. 1a

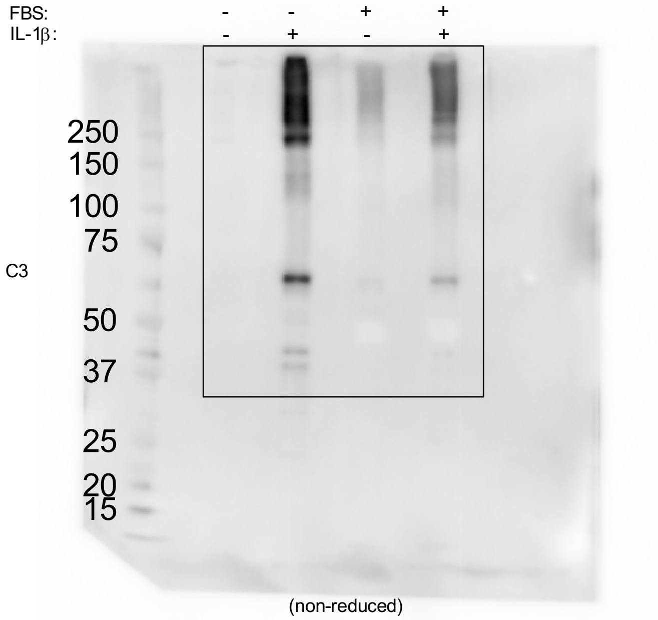

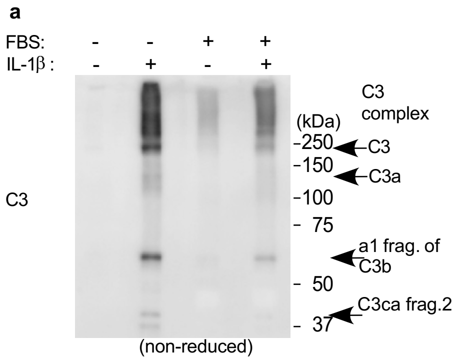

stripped and re-bloted with Factor B

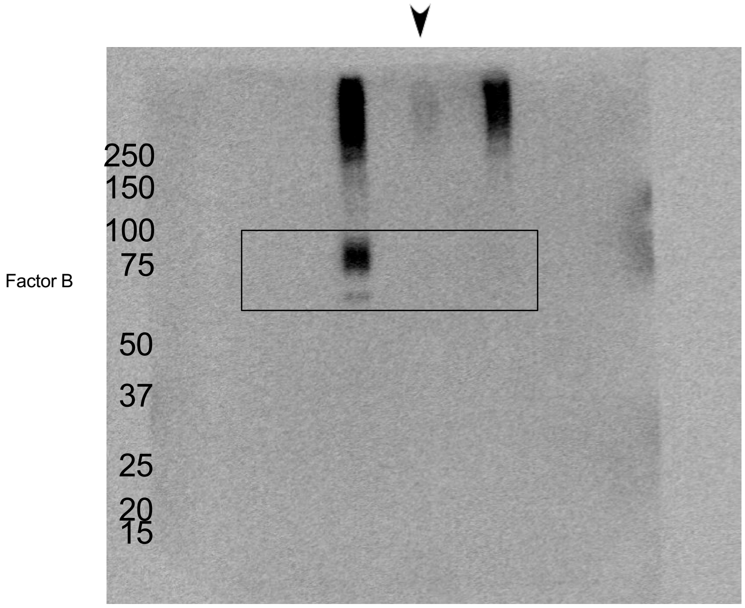

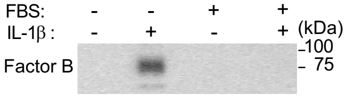

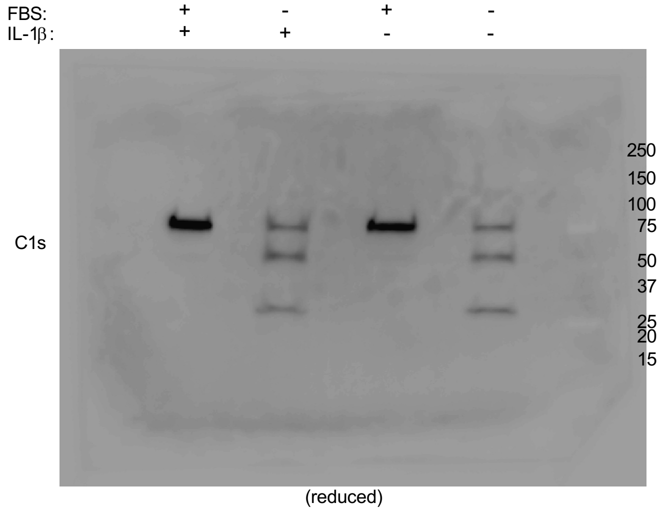

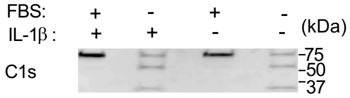

Fig. 1c

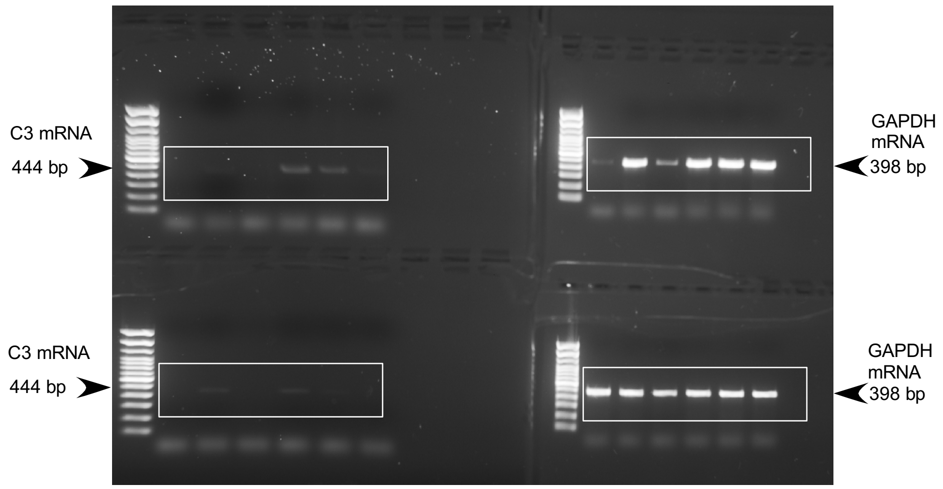

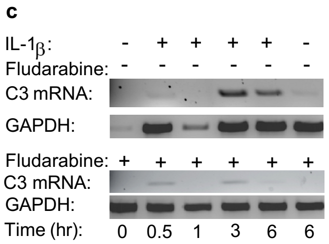

Fig. 1e

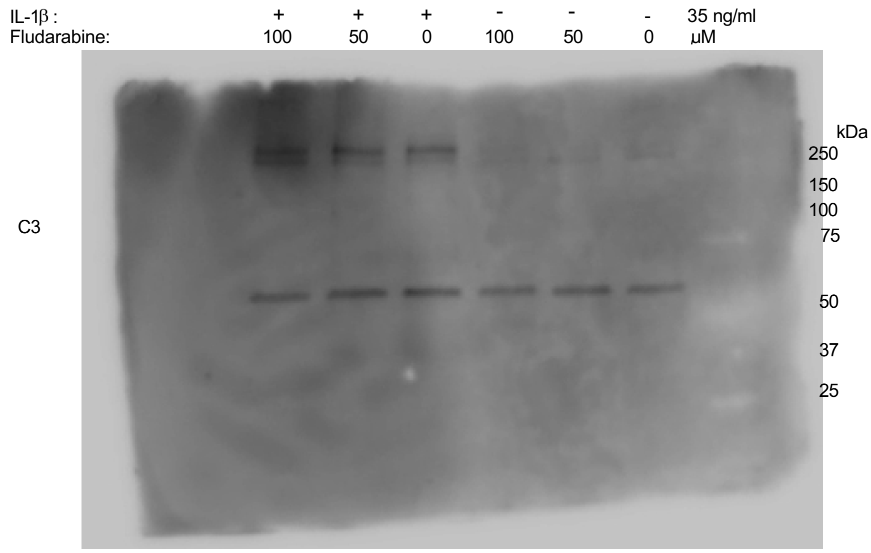

(reduced)

stripped and re-bloted with Factor B

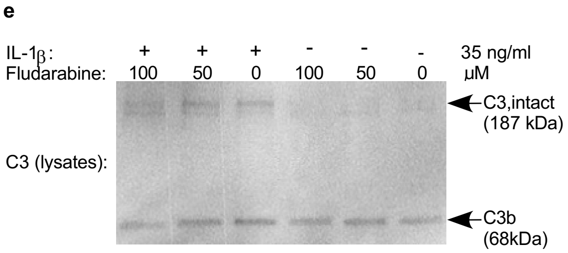

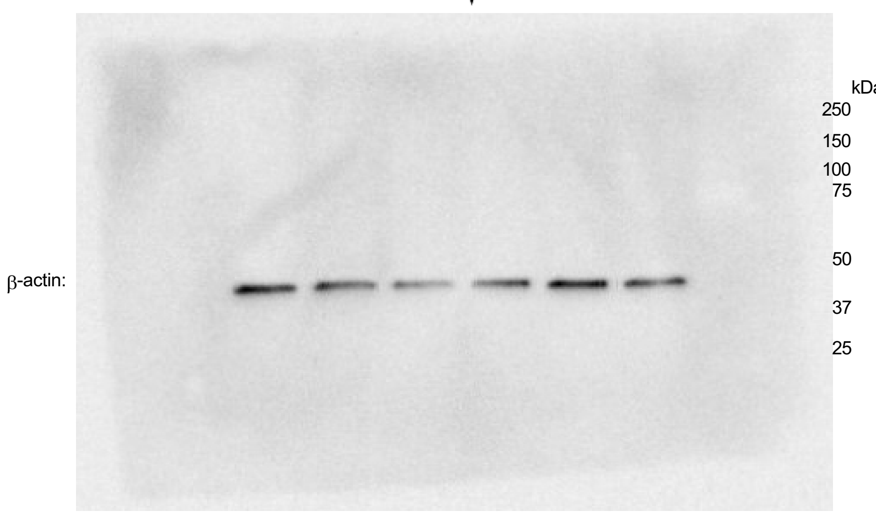

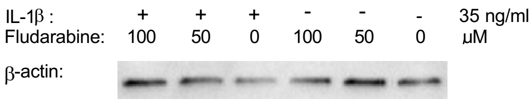

Fig. 1e

C3:
(conditioned
media)

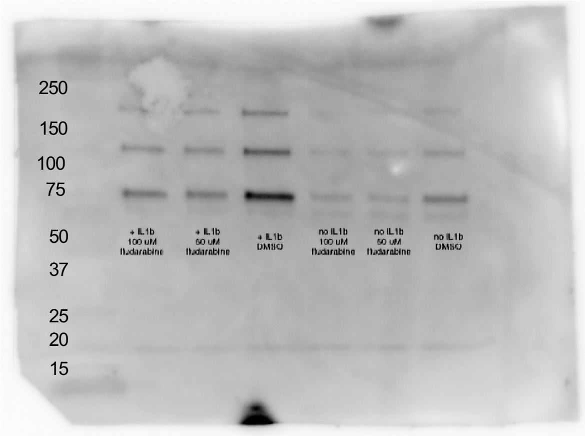

(reduced)

IL-1β :	+	+	+	-	-	-	35 ng/ml
Fludarabine:	100	50	0	100	50	0	µM

C3:
(conditioned
media)

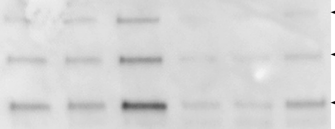

←C3,intact (187 kDa)
←C3a (115 kDa)
←C3b (68 kDa)

Factor B:

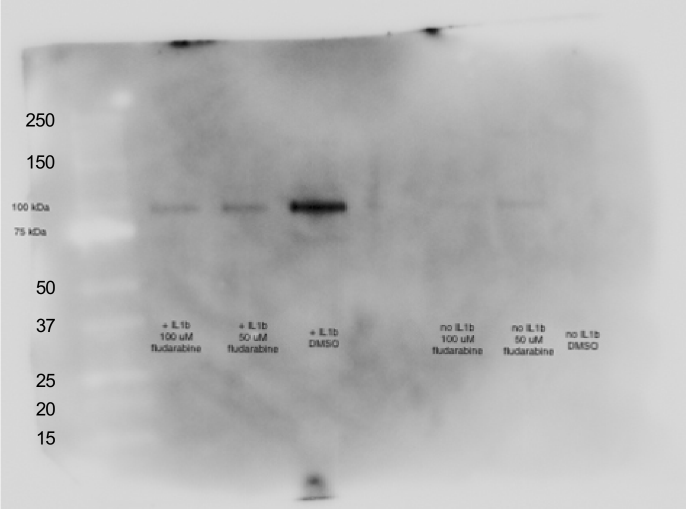

IL-1β :	+	+	+	-	-	-	35 ng/ml
Fludarabine:	100	50	0	100	50	0	µM

Factor B:

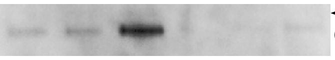

←Factor B (intact, 93kDa)

Fig. 1f

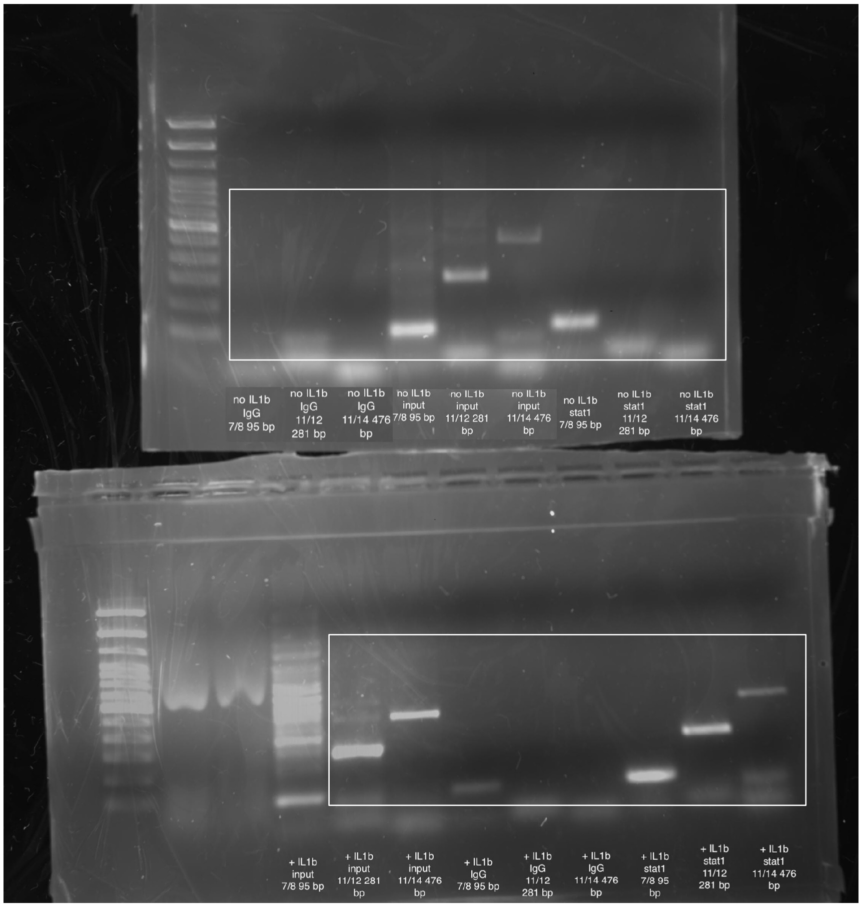
no IL1b IgG 7/8 95 bp
no IL1b IgG 11/12 281 bp
no IL1b IgG 11/14 476 bp
no IL1b input 7/8 95 bp
no IL1b input 11/12 281 bp
no IL1b input 11/14 476 bp
no IL1b stat1 7/8 95 bp
no IL1b stat1 11/12 281 bp
no IL1b stat1 11/14 476 bp
+ IL1b input 7/8 95 bp
+ IL1b input 11/12 281 bp
+ IL1b input 11/14 476 bp
+ IL1b IgG 7/8 95 bp
+ IL1b IgG 11/12 281 bp
+ IL1b IgG 11/14 476 bp
+ IL1b stat1 7/8 95 bp
+ IL1b stat1 11/12 281 bp
+ IL1b stat1 11/14 476 bp

f

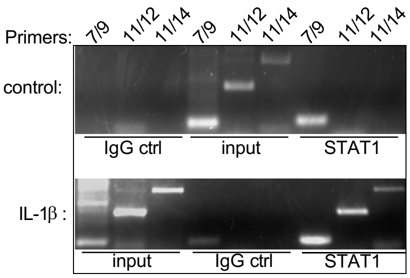
Primers: 7/9 11/12 11/14 7/9 11/12 11/14 7/9 11/12 11/14
control:
IgG ctrl
input
STAT1
IL-1β :
input
IgG ctrl
STAT1
