# Supplementary material for: Inhibition of complement C3 prevents osteoarthritis progression in guinea pigs by blocking STAT1 activation
Source: Commun Biol. 2024 Mar 27;7:370. doi: 10.1038/s42003-024-06051-6 (PMC10973449; doi:10.1038/s42003-024-06051-6)
Supplement: Supplementary file 3 — Description of Additional Supplementary Files [file 42003_2024_6051_MOESM3_ESM.docx]

**Description of Additional Supplementary Files**

**File name:** Supplementary Data 1

**Description:** list of primers used in the study.

**File name:** Supplementary Data 2

**Description:** The extracellular proteins in the supernatant from human primary chondrocytes treated with IL-1β
