# Supplementary material for: Inhibition of complement C3 prevents osteoarthritis progression in guinea pigs by blocking STAT1 activation
Source: Commun Biol. 2024 Mar 27;7:370. doi: 10.1038/s42003-024-06051-6 (PMC10973449; doi:10.1038/s42003-024-06051-6)
Supplement: Supplementary file 6 — Reporting Summary [file 42003_2024_6051_MOESM6_ESM.pdf]

Corresponding author(s): Jen X. Xu

Last updated by author(s): Nov 1, 2023

Reporting Summary

Nature Portfolio wishes to improve the reproducibility of the work that we publish. This form provides structure for consistency and transparency in reporting. For further information on Nature Portfolio policies, see our Editorial Policies and the Editorial Policy Checklist.

Statistics

For all statistical analyses, confirm that the following items are present in the figure legend, table legend, main text, or Methods section.

n/a	Confirmed	
☐	☒	The exact sample size (*n*) for each experimental group/condition, given as a discrete number and unit of measurement
☐	☒	A statement on whether measurements were taken from distinct samples or whether the same sample was measured repeatedly
☐	☒	The statistical test(s) used AND whether they are one- or two-sided<br>*Only common tests should be described solely by name; describe more complex techniques in the Methods section.*
☒	☐	A description of all covariates tested
☒	☐	A description of any assumptions or corrections, such as tests of normality and adjustment for multiple comparisons
☐	☒	A full description of the statistical parameters including central tendency (e.g. means) or other basic estimates (e.g. regression coefficient) AND variation (e.g. standard deviation) or associated estimates of uncertainty (e.g. confidence intervals)
☐	☒	For null hypothesis testing, the test statistic (e.g. *F*, *t*, *r*) with confidence intervals, effect sizes, degrees of freedom and *P* value noted<br>*Give P values as exact values whenever suitable.*
☒	☐	For Bayesian analysis, information on the choice of priors and Markov chain Monte Carlo settings
☒	☐	For hierarchical and complex designs, identification of the appropriate level for tests and full reporting of outcomes
☒	☐	Estimates of effect sizes (e.g. Cohen's *d*, Pearson's *r*), indicating how they were calculated

Our web collection on statistics for biologists contains articles on many of the points above.

Software and code

Policy information about availability of computer code

Data collection	N/A
Data analysis	N/A

For manuscripts utilizing custom algorithms or software that are central to the research but not yet described in published literature, software must be made available to editors and reviewers. We strongly encourage code deposition in a community repository (e.g. GitHub). See the Nature Portfolio guidelines for submitting code & software for further information.

Data

Policy information about availability of data

All manuscripts must include a data availability statement. This statement should provide the following information, where applicable:

- Accession codes, unique identifiers, or web links for publicly available datasets
- A description of any restrictions on data availability
- For clinical datasets or third party data, please ensure that the statement adheres to our policy

All data supporting the findings of this study are available within the paper and its supplementary information. Additional data related to this paper are available from the corresponding author on request.

Research involving human participants, their data, or biological material

Policy information about studies with human participants or human data. See also policy information about sex, gender (identity/presentation), and sexual orientation and race, ethnicity and racism.

Reporting on sex and gender	N/A
Reporting on race, ethnicity, or other socially relevant groupings	N/A
Population characteristics	N/A
Recruitment	N/A
Ethics oversight	N/A

Note that full information on the approval of the study protocol must also be provided in the manuscript.

Field-specific reporting

Please select the one below that is the best fit for your research. If you are not sure, read the appropriate sections before making your selection.

☒ Life sciences ☐ Behavioural & social sciences ☐ Ecological, evolutionary & environmental sciences

For a reference copy of the document with all sections, see nature.com/documents/nr-reporting-summary-flat.pdf

Life sciences study design

All studies must disclose on these points even when the disclosure is negative.

Sample size	No sample size was done for Dunkin-Hartley guinea pigs as the facility could only hold 18 so we did the max.
Data exclusions	no data were excluded. all samples that could be used were used.
Replication	in vitro experiments had at least 3 biological replicates.
Randomization	Guinea pigs were totally random in receiving the doses of fludarabine.
Blinding	Data collection was not blinded, but parts of analysis was.

Reporting for specific materials, systems and methods

We require information from authors about some types of materials, experimental systems and methods used in many studies. Here, indicate whether each material, system or method listed is relevant to your study. If you are not sure if a list item applies to your research, read the appropriate section before selecting a response.

Materials & experimental systems

n/a	Involved in the study	
☐	☒	Antibodies
☐	☒	Eukaryotic cell lines
☒	☐	Palaeontology and archaeology
☐	☒	Animals and other organisms
☒	☐	Clinical data
☒	☐	Dual use research of concern
☒	☐	Plants

Methods

n/a	Involved in the study	
☒	☐	ChIP-seq
☒	☐	Flow cytometry
☒	☐	MRI-based neuroimaging

Antibodies

Antibodies used	human anti-C1s (R&D, cat: AF2060SP, lot: URC012107A), human anti-Factor B (R&D systems, cat: AF2739, VIT022106A), human anti-complement 3 (Invitrogen, cat: PA129715, lot: WF3301883E), human anti-β-actin (Cell signaling, cat: 3700). Rabbit anti-sheep, HRP (Invitrogen, cat: PI31480, lot: WF3302716), Goat anti-Mouse IgG (H+L) Secondary Antibody, HRP (Invitrogen, cat: 32430, lot: VJ313743), Rabbit anti-Goat IgG (H+L) Secondary Antibody, HRP (Invitrogen, cat: 31402, lot: WF3301883E).

	IL-6 for ELISA (Biolegend,cat: 430506). STAT1 Rabbit Antibody (Catalog #: 9172S, Cell Signaling). Rabbit IgG antibody (Catalog #: 2729S, Cell Signaling).
Validation	Invitrogen antibodies were validated with immunocytochemistry as per manufacturer. R&D antibodies and Cell Signaling were validated using multiple different methods to assure accuracy. Biolegend validates antibodies for ELISA.

Eukaryotic cell lines

Policy information about cell lines and Sex and Gender in Research

Cell line source(s)	Human articular chondrocytes from the knee. Cell Applications Catalog #: 402K-05a, Lot #3373
Authentication	Cells were shipped at second passage directly and demonstrated ability to proliferate in cell media.
Mycoplasma contamination	All cells were checked and free of any fungi.
Commonly misidentified lines (See ICLAC register)	*Name any commonly misidentified cell lines used in the study and provide a rationale for their use.*

Animals and other research organisms

Policy information about studies involving animals; ARRIVE guidelines recommended for reporting animal research, and Sex and Gender in Research

Laboratory animals	Dunkin-Hartley Guinea pigs 5-months-old
Wild animals	N/A
Reporting on sex	There have been prior studies that showed male Dunkin-Hartley guinea pigs develop OA at a faster and more severe rate than the females so we chose all males for the study as it was only feasible for us to work with 18 guinea pigs at the time.
Field-collected samples	N/A
Ethics oversight	*Identify the organization(s) that approved or provided guidance on the study protocol, OR state that no ethical approval or guidance was required and explain why not.*

Note that full information on the approval of the study protocol must also be provided in the manuscript.

Plants

Seed stocks	N/A
Novel plant genotypes	N/A
Authentication	N/A
